# Airway microbial and metabolic features associated with ICS therapy in COPD

**DOI:** 10.3389/fphar.2025.1714879

**Published:** 2025-12-02

**Authors:** Lijun Chen, Mei Yang, Qixin Wang, Yan Tang, Hongxia Yu, Xianhui Luo, Xiaochao Du, Haizong Hu

**Affiliations:** 1 Department of Respiratory and Critical Care Medicine, Dazhou Hospital of Integrated Traditional Chinese and Western Medicine/Dazhou Second People’s Hospital, Dazhou, China; 2 Department of Laboratory Medicine, Deyang People’s Hospital, Deyang, China

**Keywords:** COPD, inhaled corticosteroids, airway microbiome, metabolomics, caffeine metabolism, methylxanthines, host–microbe interactions

## Abstract

**Background:**

Inhaled corticosteroids (ICS) are a cornerstone of therapy for selected phenotypes of chronic obstructive pulmonary disease (COPD), yet the underlying mechanisms remain incompletely understood. Increasing evidence suggests that airway microbiome and their metabolites play crucial roles in shaping host immune responses and disease progression.

**Objective:**

This study used multi-omics technology to explore the differences in sputum microbiome, metabolites and the systematic connections in patients with stable COPD who use or not ICS.

**Methods:**

We performed an integrated microbiome–metabolome analysis of induced sputum samples from 53 stable COPD patients (40 ICS users and 13 non-users). Microbial communities were profiled using 16S rRNA sequencing, while metabolic signatures were assessed via liquid chromatography–mass spectrometry. Correlation analyses were conducted to explore microbe–metabolite interactions.

**Results:**

The microbial alpha diversity (Simpson, Shannon, Pielou indices; P < 0.05) was significantly reduced in the ICS group, and the beta diversity was distincted between the two groups. The relative abundance of *Firmicutes*, *Streptococcus* was significantly reduced, and the relative abundance of *Veillonella* was significantly increased in the ICS group (P < 0.05). Metabolomic profiling identified 70 differential metabolites enriched in pathways including caffeine metabolism, cobalamin transport and metabolism, and cysteine/methionine metabolism. Notably, *Streptococcus* abundance was negatively correlated with methylxanthine intermediates (caffeine, theobromine, 1,7-dimethylxanthine, 1-methylxanthine), while Veillonella abundance showed positive correlations with these metabolites (P < 0.05).

**Conclusion:**

Our findings suggest that ICS therapy not only reshapes the airway microbial ecosystem but also alters host–microbe co-metabolic pathways, particularly caffeine metabolism. By reducing microbial degradation of methylxanthines, ICS may enhance the bioavailability of bronchodilatory compounds, providing a potential microbiome-mediated adjunctive mechanism of action. These insights advance our understanding of ICS pharmacology in COPD and highlight the therapeutic potential of targeting microbiome–metabolite interactions.

## Graphical Abstract

**Figure:**
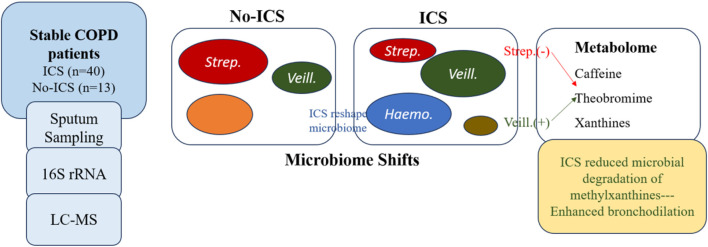
Inhaled corticosteroids (ICS) reshape the airway microbiome and alter host–microbiome -metabolism in stable COPD.ICS reduces *Streptococcus* and enriches *Veillonella* and *Haemophilus*, accompanied by changes in caffeine metabolism. Reduced microbial degradation of methylxanthines may prolong bronchodilatory effects, revealing a microbiome-mediated mechanism of ICS action.

## Background

Chronic obstructive pulmonary disease (COPD) is a prevalent respiratory disorder characterized by chronic dyspnea, cough, and sputum production. COPD has a high incidence and mortality, posing a substantial social and economic burden. Patients often experience premature death due to acute exacerbations or comorbidities. In 2015, COPD accounted for 3.2 million deaths worldwide (Viegi et al., 2020), representing 5.7% of all-cause mortality (Collaborators, 2020). In China, COPD is become the third leading cause of death (Zhou et al., 2019), with nearly 100 million affected individuals and a rising prevalence in adults ≥40 years of age (Wang et al., 2018). The annual *per capita* healthcare costs for COPD in China are substantial, with longer hospitalization durations compared to Western countries (Anees et al., 2020). Owing to risk factors such as smoking, dust, air pollution, and aging, the global health burden of COPD is projected to continue rising in the coming decades (Mathers and Loncar, 2006).

Inhaled corticosteroids (ICS) are among the most widely used anti-inflammatory therapies for COPD. They effectively reduce exacerbation frequency, improve lung function, enhance exercise tolerance, and improve quality of life (Lea et al., 2023). Although COPD has historically been regarded as a “corticosteroid-resistant” disease, the GOLD guidelines (Global Initiative for Chronic Obstructive Lung Disease, 2025) recommend ICS for selected phenotypes, particularly patients with a high risk of exacerbation and eosinophilic inflammation. ICS modulate eosinophilic inflammation by inducing eosinophil apoptosis, but may also prolong neutrophil survival, thereby aggravating neutrophilic inflammation (Singh et al., 2020). Furthermore, ICS exert multiple anti-inflammatory effects on airway epithelial cells, including suppression of pro-inflammatory cytokines (IL-1β, GM-CSF, IL-8), downregulation of iNOS and COX-2, and inhibition of adhesion molecule expression, collectively attenuating airway inflammation (Barnes, 2010).

Airway inflammation is central to COPD pathogenesis and progression, and airway microbiota shifts are closely linked to disease advancement and exacerbations (Mammen and Sethi, 2016). Owing to their immunosuppressive effects, ICS can alter host–microbe interactions, thereby reshaping the airway microbiome. Indeed, ICS use has been associated with microbiome alterations in asthma and sinusitis (Turturice et al., 2017; Ramakrishnan et al., 2018). Microbial communities, through metabolites, structural components, and toxins, regulate innate and adaptive immune responses and influence pulmonary inflammation (Cait et al., 2018; Spacova et al., 2019). Metabolites, in particular, play pivotal roles in shaping host physiology and immunity, and are closely tied to inflammatory processes (Lavelle and Sokol, 2020). Previous multi-omics analyses suggest that the lower airway microbiome influences COPD by producing and releasing bioactive metabolites (Wang et al., 2020). However, studies investigating microbiome functional features and microbe–metabolite interactions in relation to ICS therapy remain scarce. Understanding the impact of ICS on the lower airway microenvironment is critical for elucidating therapeutic mechanisms and guiding personalized interventions.

## Methods

All stable COPD patients were recruited from the Department of Respiratory and Critical Care Medicine. This study was approved by the institutional ethics committee, and written informed consent was obtained from all participants. Clinical data were extracted from the COPD clinical research database. We recruited 53 patients with stable COPD from a clinical research database and outpatient clinic, including 40 ICS users and 13 non-ICS users. Inclusion criteria were stable COPD patients aged 30–80 years. Exclusion criteria included comorbid respiratory diseases, severe systemic disorders, or malignancies. Demographic and clinical characteristics, including age, sex, body mass index (BMI), smoking history, comorbidities, disease duration, and exacerbation frequency, were collected. Symptom and quality-of-life scores (modified British Medical research council (mMRC), COPD assessment test (CAT), The St George’s Respiratory Questionnaire (SGRQ), Clinical COPD Questionnaire (CCQ), Hospital anxiety and depression scale (HADS)) were assessed, along with lung function indices (FEV1, FEV1%, FVC, FVC%, FEV1/FVC). Owing to clinical conditions or patient preference, spirometric assessments were not completed for all participants, leading to partial missingness in pulmonary function data. Based on the available measurements, the distribution of GOLD stages was as follows: GOLD II (moderate), n = 10; GOLD III (severe), n = 27; and GOLD IV (very severe), n = 12. Patients with GOLD I (mild) COPD were not included in the cohort.

Induced sputum samples were collected to assess the airway microbiome and metabolome. This approach was chosen because it is non-invasive, reproducible, and clinically feasible for stable COPD patients, while bronchoalveolar lavage fluid (BALF) collection is invasive and not ethically suitable in non-exacerbating subjects. Previous studies have demonstrated that induced sputum reliably reflects the microbial composition of the lower airways (Huang et al., 2011; Pragman et al., 2012). To minimize upper airway or oral contamination, participants rinsed their mouths thoroughly before sputum induction. Viable, non-viable, and inflammatory cells were quantified in the sputum samples. Samples exhibiting a cell viability of less than 40% or containing more than 50% squamous epithelial cells were excluded from subsequent analyses. Samples exceeding this threshold were excluded. Induced sputum samples were processed for 16S rRNA sequencing and metabolomics analysis (LC-MS in both positive and negative ion modes). Genomic DNA was extracted using the QIAamp DNA Mini Kit (Qiagen, Germany). The V4 region of the bacterial 16S rRNA gene was amplified with primers 515F/806R. PCR products were purified and sequenced on an Illumina NovaSeq 6000 platform. Sputum samples were thawed on ice and homogenized using a vortex mixer. For hydrophilic metabolite extraction, 50 μL of each sample was mixed with 300 μL of 20% acetonitrile–methanol extraction solution containing internal standards, vortexed for 3 min, and centrifuged at 12,000 rpm for 10 min at 4 °C. Two hundred microliters of the supernatant was collected, incubated at −20 °C for 30 min, and centrifuged again under the same conditions. An aliquot of 18 μL of the resulting supernatant was transferred to autosampler vials for LC–MS analysis. For hydrophobic metabolite extraction, 50 μL of each sample was mixed with 1 mL of lipid extraction solution (methyl tert-butyl ether:methanol = 3:1, v/v) containing internal standards, vortexed for 15 min, followed by the addition of 200 μL of distilled water and vortexed for another minute. After centrifugation at 12,000 rpm for 10 min at 4 °C, 200 μL of the upper phase was collected, evaporated to dryness, reconstituted in 200 μL of mobile phase B, vortexed for 3 min, and centrifuged again before injection. Chromatographic separation of hydrophilic compounds was performed on a Waters ACQUITY UPLC HSS T3 column (1.8 μm, 2.1 × 100 mm) with 0.1% formic acid in water as mobile phase A and acetonitrile as mobile phase B, under a gradient elution program (0–11 min, 95%–10% A; 11–12 min, 10% A; 12–12.1 min, 10%–95% A; 12.1–14 min, 95% A). The flow rate was 0.4 mL/min, injection volume 2 μL, and column temperature 37 °C. Hydrophobic compounds were separated using a Thermo Accucore C30 column (2.6 μm, 2.1 × 100 mm) with mobile phase A (60% acetonitrile and 40% water containing 0.1% formic acid and 10 mM ammonium formate) and mobile phase B (acetonitrile/isopropanol, 10:90, v/v). The gradient elution program was 0–2 min, 80%–70% A; 2–4 min, 70%–40% A; 4–9 min, 40%–15% A; 9–14 min, 15%–10% A; 14–15.5 min, 10%–5% A; 15.5–17.3 min, 10%–5% A; 17.3–20 min, 5%–80% A. The flow rate was 0.35 mL/min, injection volume 2 μL, and column temperature 45 °C. Mass spectrometry was performed using an electrospray ionization (ESI) source, with ion source gases I and II set at 55 and 60 psi, curtain gas at 25 psi, temperature 500 °C, and ion spray voltages of +5500 V (positive mode) and −4500 V (negative mode) for hydrophilic metabolites, and 45 and 55 psi, 35 psi curtain gas, 500 °C, +5500/−4500 V for hydrophobic metabolites, respectively. Raw LC–MS/MS data were processed and analyzed using Analyst 1.6.3 software (AB Sciex, Framingham, MA, USA) for metabolite identification and quantification. Bioinformatics pipelines included OTU clustering, taxonomic annotation, alpha/beta diversity analysis, differential taxa identification (Wilcoxon/Kruskal–Wallis, LEfSe, Random Forest), and correlation analysis (Spearman). Alpha diversity indices were calculated as follows:

$$\text{Shannon diversity index}:H=-\sum \left(pᵢ\times \ln\left(pᵢ\right)\right)$$

$$\text{Simpson diversity index}:D=1-\sum \left(p{ᵢ}^{2}\right)$$

$$\text{Pielou}’s\text{ evenness index}:J=H\;/\;\ln\left(S\right)$$

where pᵢ represents the relative abundance of species i, and S denotes the total number of observed species. A higher Shannon or Simpson index indicates greater species diversity, while Pielou’s evenness reflects the uniformity of community composition. Alpha diversity indices (Shannon, Simpson, Pielou, Observed, Chao1) were computed. Group differences were tested using Welch’s t-test and Cohen’s d reported as effect size. Bray-Curtis dissimilarity and PERMANOVA (999 permutations) were used for beta-diversity; balanced subsampling PERMANOVA (200 iterations) was performed to mitigate unequal group sizes. Metabolomics data were log1p transformed, imputed with column-wise mean, standardized, and analyzed by PCA (R^2^ and Q^2^ reported). Differential metabolites were tested by t-tests with Benjamini–Hochberg FDR correction. Metabolites were screened (VIP ≥1, |log2FC| ≥ 1, P < 0.05) and subjected to KEGG enrichment analysis. Statistical analyses were performed using R (v4.1.3) and GraphPad Prism 9.0.

## Results

The two groups showed no significant differences in baseline demographics or lung function (P > 0.05), although the CAT scores were higher in the ICS group (P = 0.023) (Tables 1, 2), indicating that patients using ICS had a greater symptom burden. The full list of ICS medications has been added, including budesonide, fluticasone, along with their dose. No patients were on ICS during sampling. Details are included in Supplementary Table S1. The Supplementary Table S2 has been listed all concomitant medications (bronchodilators, statins, antihypertensives, antidiabetics).

**TABLE 1:** Baseline demographic and clinical characteristics of COPD patients in ICS and No-ICS groups.

Items	ICS (n = 40)	No-ICS (n = 13)	P value
Age (years)*	63.90 ± 8.74	64.29 ± 6.43	0.866
Sex, male/female	42/10	15/2	0.716
BMI (kg/m^2^)*	22.89 ± 3.60	24.64 ± 2.14	0.081
Smoking history, N (%)			
Current smoking	14 (26.9)	4 (23.5)	0.782
Smoked in the past	23 (44.2)	10 (58.8)	0.403
No smoking	15 (28.9)	3 (16.7)	0.361
Smoking index (package* years)^#^	27.5 (0–40)	35.0 (12.3–42.5)	0.438
Disease duration, (year)^#^	4 (1–7)	2.5 (1–3)	0.189
Number of exacerbations in the past year	1 (0–2.8)	0.5 (0–1)	0.668
Number of hospitalizations in the past year	0 (0–2)	0 (0–1)	0.437

*: Mean ± SD; #: Median (IQR), and P value in bold indicates a statistically significant difference. Summary of age, sex, body mass index (BMI), smoking history, disease duration, number of exacerbations and hospitalizations in the past year, and comorbidities. No significant differences were observed between ICS (n = 40) and No-ICS (n = 13) groups (P > 0.05).

**TABLE 2:** Lung function and symptom scores in COPD patients in ICS and No-ICS groups.

Items	ICS (n = 40)	No-ICS (n = 13)	P value
FEV1 (L)	1.11 ± 0.45	1.30 ± 0.63	0.012
FEV1%	45.96 ± 16.09	59.13 ± 15.26	0.259
FVC (L)	2.40 ± 0.66	2.99 ± 1.21	0.427
FVC %	80.99 ± 9.46	86.50 ± 16.55	0.534
FEV1/FVC (%)	58.24 ± 6.11	49.98 ± 8.17	0.325
mMRC score	1.60 ± 0.82	1.41 ± 1.06	0.514
CAT score	8.75 ± 5.58	13.00 ± 5.48	**0.023**
SGRQ score	23.60 ± 8.52	29.15 ± 12.50	0.338
CCQ score	16.63 ± 4.47	18.08 ± 4.61	0.412
HADS score	16.63 ± 4.07	17.88 ± 4.21	0.251

The data is described as Mean ± SD, and P value in bold indicates a statistically significant difference. Comparison of pulmonary function indices (FEV1, FEV1%, FVC, FVC%, FEV1/FVC) and symptom-related scores (mMRC, CAT, SGRQ, CCQ, HADS) between ICS, and No-ICS, groups; CAT, scores were significantly higher in the ICS, group compared with the No-ICS, group (P = 0.023); no other significant differences were observed.

### Microbiome

**FIGURE 1:**
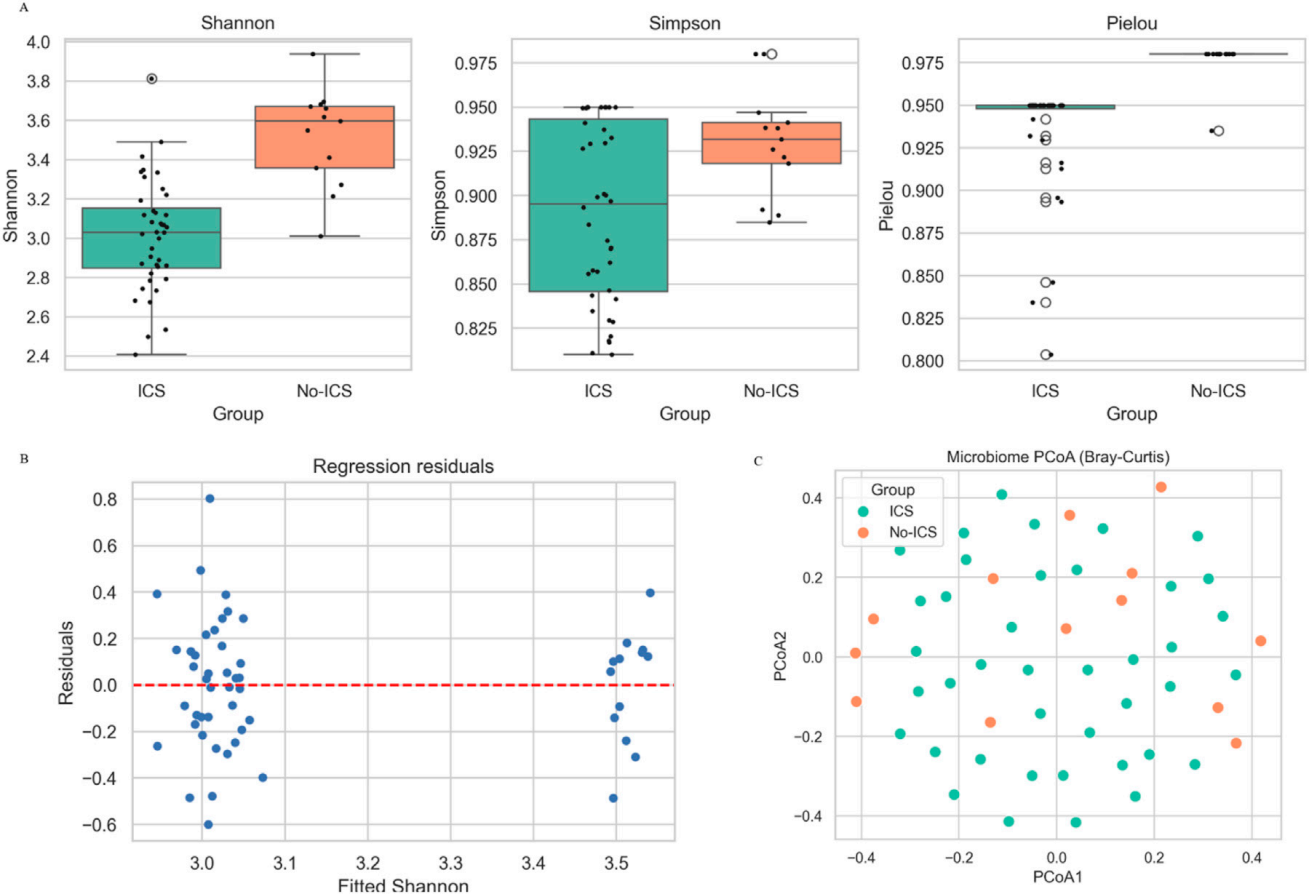
Effects of ICS on sputum microbial diversity in COPD patients. **(A)** Alpha diversity indices (Shannon, Simpson, Pielou’s evenness, Observed richness, and Chao1) were significantly lower in the ICS group compared with the No-ICS group (P < 0.05). **(B)** Regression residuals. **(C)** Principal coordinates analysis (PCoA) based on Bray–Curtis distance between ICS and No-ICS groups.

Across all five α-diversity indices (Shannon, Simpson, Pielou’s evenness, Observed richness, and Chao1), ICS users demonstrated significantly lower diversity than non-ICS users (all P < 0.05; Figure 1A; Supplementary Tables S3, S4), indicating that there were significant differences in the richness and uniformity of microbiome between the two groups. Effect size estimates (Cohen’s d) were consistently in the moderate-to-large range (−0.85–2.46), indicating robust biological differences. These findings remained significant after adjustment for FEV1% predicted and CAT score (multivariable linear regression, all adjusted P < 0.05). A summary of the regression model output is provided in Figure 1B; Supplementary Table S5. Principal coordinate analysis (PCoA) based on Bray–Curtis dissimilarity demonstrated a distinct separation between groups (PERMANOVA: R^2^ = 0.133, P = 0.001) (Figure 1C), indicating that ICS significantly altered the composition of the microbial community. To account for unequal sample sizes (40 vs. 13), we additionally performed 200 iterations of balanced-subset PERMANOVA (43 vs. 13 randomly sampled). ICS remained significant in all iterations (mean P = 0.046), supporting the robustness of group differences (Supplementary Table S6).

**FIGURE 2:**
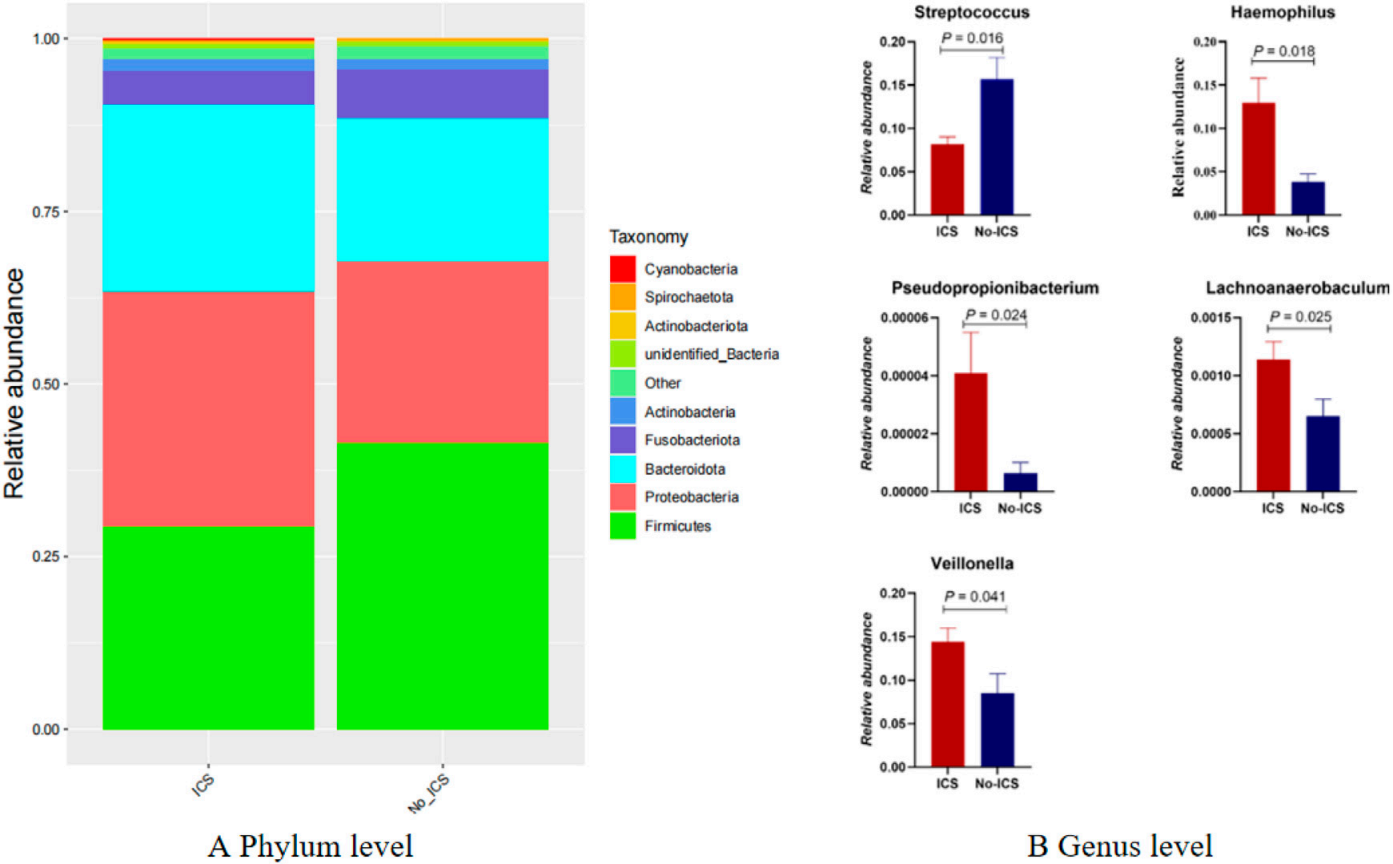
Taxonomic differences in airway microbiome between ICS and No-ICS groups. **(A)** Relative abundance of dominant phyla, showing reduced *Firmicutes* in the ICS group (P < 0.05). **(B)** Differentially abundant genera between groups, with enrichment of *Streptococcus* and *Pseudopropionibacterium* in the No-ICS group, and enrichment of *Lachnoanaerobaculum*, *Veillonella*, and *Haemophilus* in the ICS group (P < 0.05).

At the phylum level, the relative abundance *of Firmicutes* was reduced in the ICS group (P < 0.05) (Figure 2A). At the genus level, *Streptococcus* and *Pseudopropionibacterium* were enriched in the No-ICS group, while *Veillonella*, *Haemophilus*, and *Lachnoanaerobaculum* were enriched in the ICS group (Figure 2B).

**FIGURE 3:**
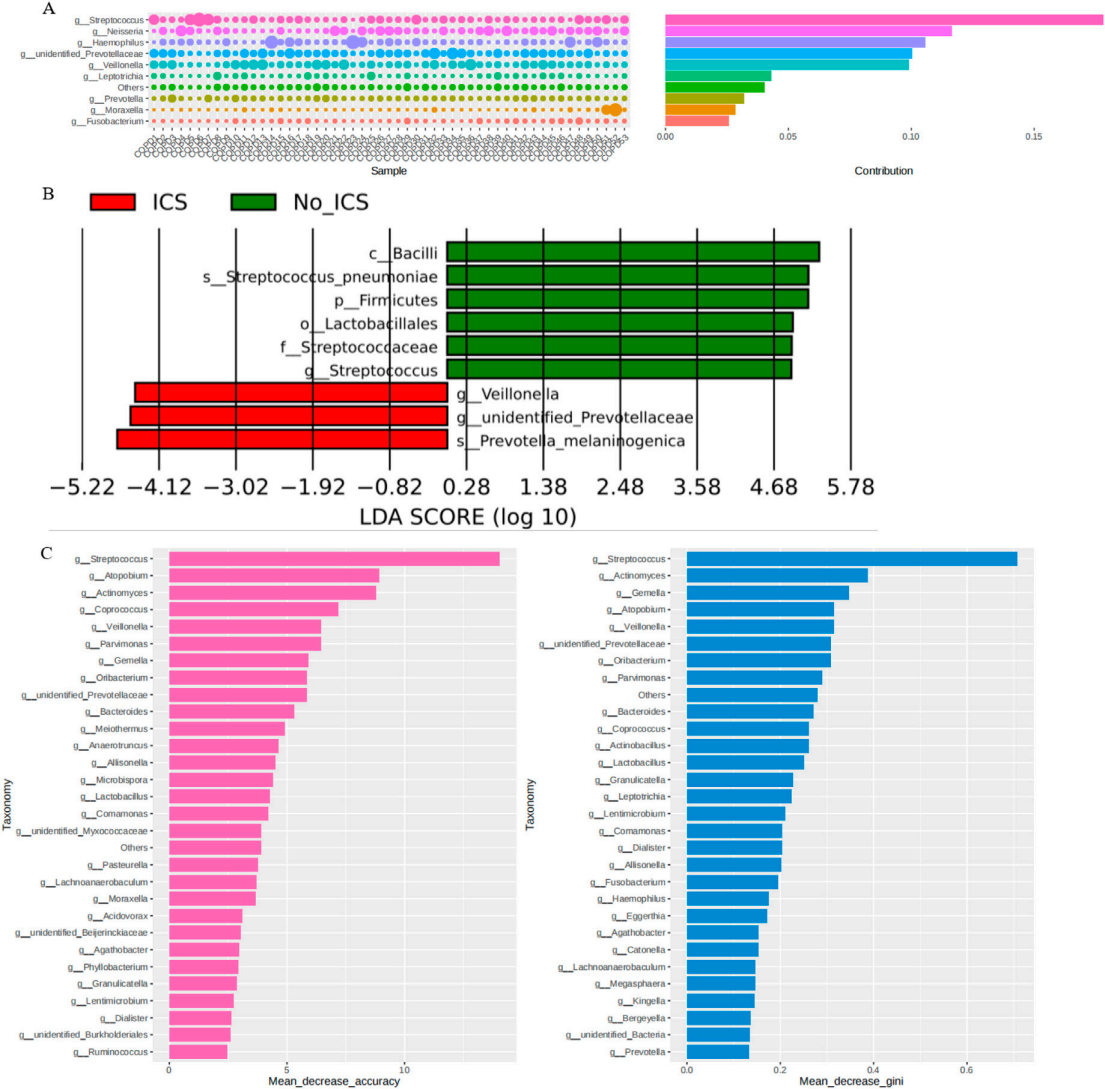
Discriminatory taxa and their contribution to group differences. **(A)** Simper analysis at the genus level shsowing the contribution of *Streptococcus* to intergroup variation. **(B)** Linear discriminant analysis (LDA) effect size (LEfSe) indicating taxa enriched in ICS versus No-ICS groups. **(C)** Random Forest analysis ranking taxa by importance for group discrimination, highlighting *Streptococcus* as the most influential genus.

The Simper analysis at the genus level showed the contribution of *Streptococcus* to the difference between the two groups (Figure 3A). The LDA distribution histogram (Figure 3B) shows that *Veillonella*, *unidentified Prevotellaceae*, and *Prevotella-melaninogenica* were significantly enriched in the ICS group (P < 0.05); *Bacilli*, *Streptococcus-pneumoniae*, *Firmicutes*, *Lactobacillales*, *Streptococcaceae* and *Streptococcus* were significantly enriched in the No-ICS group (P < 0.05). The results of random forest analysis also suggested that the *Streptococcus* was of the greatest significance in terms of differences between groups (Figure 3C). LEfSe and Random Forest consistently highlighted *Streptococcus* as a major contributor to intergroup differences.

### Metabolome

**FIGURE 4:**
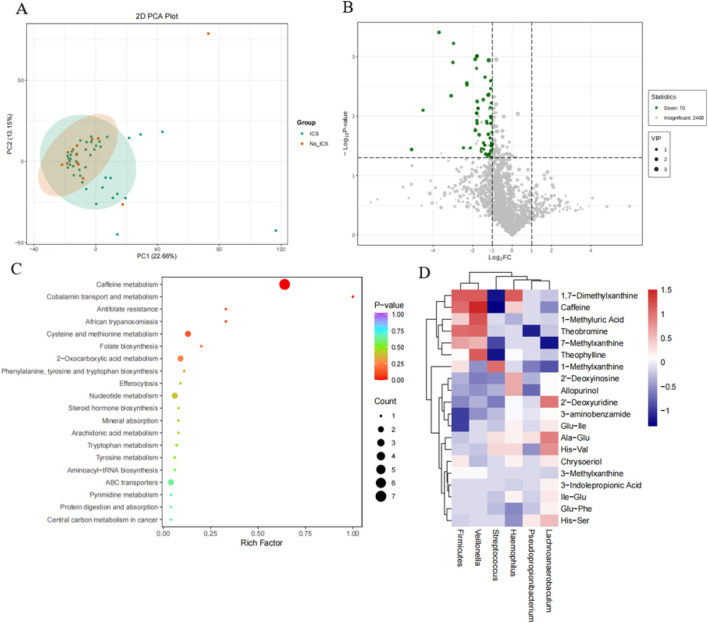
Metabolomic differences and microbiome–metabolite correlations. **(A)** Principal component analysis (PCA) of metabolomic profiles showing clear separation between ICS and No-ICS groups. **(B)** Volcano plot of 70 differentially expressed metabolites (VIP ≥1, |log2FC| ≥ 1, P < 0.05), predominantly downregulated in the ICS group. **(C)** KEGG pathway enrichment of differential metabolites, highlighting caffeine metabolism, cobalamin transport and metabolism, antifolate resistance, and cysteine/methionine metabolism. **(D)** Heatmap of Spearman correlations between top 20 differential metabolites and key microbial taxa. Negative correlations observed between *Streptococcus* and methylxanthines (caffeine, theobromine, 1,7-dimethylxanthine, 1-methylxanthine), whereas *Veillonella* exhibited positive correlations with these metabolites (P < 0.05).

Principal component analysis (PCA) revealed partial but not complete separation between the ICS and No-ICS groups, indicating mild yet observable compositional differences in sputum metabolites (Figure 4A). The PCA model exhibited R^2^ = 0.58 and Q^2^ = 0.42, suggesting a good model fit and acceptable predictive performance, consistent with metabolomics reporting standards (Worley et al., 2016). Seventy differential metabolites were identified, predominantly downregulated in ICS group (Figure 4B). These metabolites were significantly enriched in caffeine metabolism, cobalamin transport and metabolism, antifolate resistance, and cysteine/methionine metabolism (Figure 4C). Correlation analysis showed that *Streptococcus* was negatively associated with caffeine, theobromine, 1,7-dimethylxanthine, and 1-methylxanthine, while *Veillonella* abundance was positively associated with these metabolites, and Firmicutes was positively associated with 1,7-Dimethylxanthine, caffeine, theobromine; *Pseudopropionibacterium* was negatively associated with theobromine; *Lachnoanaerobaculum* was positively associated with 7-Methylxanthine; *Haemophilus* was positively associated with1,7-Dimethylxanthine (P < 0.05) (Figure 4D).

## Discussion

This study provides integrated microbiome and metabolome evidence that ICS therapy reshapes the airway microenvironment in patients with stable COPD. Our findings highlight two major insights: (i) ICS alters the taxonomic composition of the airway microbiome, reducing *Streptococcus* and enriching genera such as *Veillonella* and *Haemophilus*; and (ii) ICS profoundly influences metabolic pathways, particularly caffeine metabolism, with potential microbe–metabolite correlations Together, these results suggest that ICS exerts therapeutic effects not only through classical anti-inflammatory mechanisms (Lea et al., 2023) but also may via modulation of host–microbiome metabolic interactions.

The enrichment of *Veillonella* and *Haemophilus* in ICS group is consistent with prior observations that corticosteroid therapy may reshape the airway niche, favoring specific commensal and opportunistic taxa (Garcha et al., 2012; Contoli et al., 2017; Wang et al., 2016). Conversely, the reduction of *Streptococcus* is notable given its dual role as both a common airway commensal and a potential pathogen (Pragman et al., 2012). Importantly, our correlation analysis revealed that the relative abundance of *Streptococcus* was negatively associated with multiple methylxanthines, including caffeine, theobromine, and their intermediates. This suggests that *Streptococcus* may participate in microbial metabolism of methylxanthines (Muehlbacher et al., 2021), thereby influencing their local bioavailability. Given that caffeine and theophylline are known non-specific phosphodiesterase inhibitors with bronchodilatory properties (Muehlbacher et al., 2021), reduced microbial degradation of these compounds under ICS therapy could indirectly prolong or enhance their therapeutic effects. This may represent a novel microbiome-mediated adjunctive mechanism of ICS action.

The observed alterations in cobalamin metabolism and sulfur amino acid pathways further suggest that ICS may impact broader host–microbe metabolic networks (Higham et al., 2020; Provost et al., 2019). These pathways are linked to oxidative stress, redox balance, and immune regulation—processes central to COPD pathophysiology (Paulin et al., 2020; Cao et al., 2024; Duan et al., 2022). For instance, disruption of methionine and cysteine metabolism may alter glutathione biosynthesis and antioxidant defenses, thereby influencing disease progression (Duan et al., 2022). Such findings underscore the importance of considering not only microbial composition but also metabolic functionality when evaluating ICS effects.

Our study has limitations. The cross-sectional design precludes causal inference, and the relatively modest sample size may limit statistical power. Induced sputum samples, while reflective of the lower airway environment, may be contaminated by upper airway flora. Although minor contamination from the upper respiratory tract cannot be completely excluded, both groups underwent identical sampling and processing procedures, minimizing the potential for systematic bias. Although the ICS and No-ICS groups differed in sample size, several layers of statistical validation-including effect size quantification, balanced-subset PERMANOVA, and multivariable regression-confirmed that ICS remained independently associated with microbial and metabolic alterations. Importantly, balanced PERMANOVA (200 iterations) reproduced significant ICS effects in every iteration, indicating that the observed differences are not artifacts of unequal sampling. These analyses increase the robustness and generalizability of our findings. Future studies with larger balanced cohorts are warranted. Finally, functional predictions remain indirect; metagenomic and metatranscriptomic approaches, combined with experimental validation, will be necessary to confirm specific microbial contributions to metabolite production and degradation.

## Significance statement

Inhaled corticosteroids (ICS) are widely prescribed for chronic obstructive pulmonary disease (COPD), yet their mechanistic impact beyond direct anti-inflammatory effects remains unclear. By integrating airway microbiome and metabolome analyses, we show that ICS therapy profoundly reshapes microbial communities and metabolic pathways in stable COPD patients. ICS use reduced microbial diversity, decreased *Streptococcus*, and enriched *Veillonella* and *Haemophilus*. These microbial shifts coincided with altered metabolite profiles, particularly within caffeine metabolism. Strikingly, *Streptococcus* was inversely correlated with methylxanthine intermediates (e.g., caffeine, theobromine), suggesting that ICS may indirectly prolong the bioavailability of bronchodilatory compounds through microbiome-mediated mechanisms. These findings uncover a previously unrecognized dimension of ICS pharmacology, highlighting the airway microbiome as both a therapeutic target and a modulator of drug response in COPD.

## Data Availability

The data presented in the study are deposited in the Github, https://github.com/Yangmeimeimei/my_research_data. Further inquiries can be directed to the corresponding author.
